# Parkinson’s disease risk enhancers in microglia

**DOI:** 10.1016/j.isci.2024.108921

**Published:** 2024-01-17

**Authors:** Alix Booms, Steven E. Pierce, Edwin J.C. van der Schans, Gerhard A. Coetzee

**Affiliations:** 1 Center for Neurodegenerative Science, Van Andel Institute, Grand Rapids, MI 49503, USA; 2 Van Andel Institute graduate student, Grand Rapids, MI 49503, USA

**Keywords:** Natural sciences, Biological sciences, Physiology, Pathophysiology, Neuroscience, Molecular neuroscience, Cellular neuroscience

## Abstract

Genome-wide association studies have identified thousands of single nucleotide polymorphisms that associate with increased risk for Parkinson’s disease (PD), but the functions of most of them are unknown. Using assay for transposase-accessible chromatin (ATAC) and H3K27ac chromatin immunoprecipitation (ChIP) sequencing data, we identified 73 regulatory elements in microglia that overlap PD risk SNPs. To determine the target genes of a “risk enhancer” within intron two of *SNCA*, we used CRISPR-Cas9 to delete the open chromatin region where two PD risk SNPs reside. The loss of the enhancer led to reduced expression of multiple genes including *SNCA* and the adjacent gene *MMRN1*. It also led to expression changes of genes involved in glucose metabolism, a process that is known to be altered in PD patients. Our work expands the role of *SNCA* in PD and provides a connection between PD-associated genetic variants and underlying biology that points to a risk mechanism in microglia.

## Highlights

• 73 functional candidate risk loci were identified in microglia
• Two PD risk SNPs reside in an intragenic microglia enhancer in *SNCA*
• Deletion of the enhancer led to decreased *SNCA* and *MMRN1* expression
• Additional potential target genes were up-/downregulated by the enhancer deletion

## Graphical abstract

**Figure:**
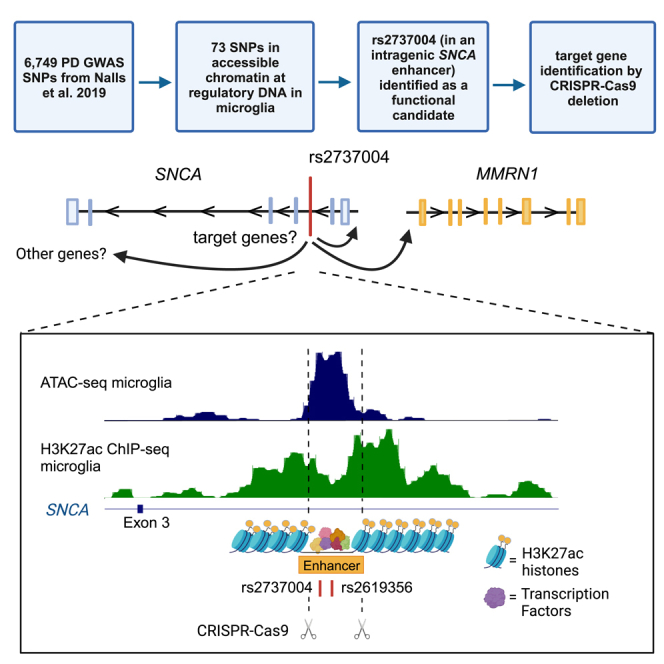

## Introduction

Genetic studies suggest that a significant portion of Parkinson’s disease (PD) risk is heritable (estimated to be up to 36%).^1^ Unlike some rare disorders caused by highly penetrant mutations in a small number of genes, PD genome-wide association studies (GWASs) have uncovered thousands of low-penetrance single-nucleotide polymorphisms (SNPs) with more modest influences on disease risk. It is hypothesized that their small effects play a role in PD predisposition through subtle changes in gene expression over the course of an entire lifespan. There are thus ongoing efforts to understand the effects of these (and other) DNA risk variants prior to disease onset.

The findings of GWASs have been challenging to interpret as not all PD-associated SNPs are biologically functional in relevant cellular and developmental contexts. Moreover, most of them are in non-coding DNA.^2^ Some variants within enhancers and promoters are known to influence gene expression regulation.^3^ However, the genes whose expression they target are not immediately apparent.^4^ Thus, the first challenges in the field are to 1) determine *which* SNPs are imposing PD risk by altering biology and 2) identify *mechanisms* by which each allele of a risk SNP leads to biological differences.

A large portion of PD risk SNPs are enriched in regulatory enhancers and promoters across multiple cell types, implying that PD susceptibility may in part be attributed to genetic variation that impacts the regulation of cell-type-specific genes and cellular processes.^5^ Microglia are the resident macrophages in the brain and are known contributors to neuroinflammation in PD.^6^ PD-associated variants reside in regulatory elements or are in and around PD-associated genes in these cells.^7^^,^^8^ However, the variants that are functional and how they influence microglia processes are largely unknown. It has been demonstrated that SNPs within regulatory elements alter transcription factor binding and gene expression.^3^^,^^9^^,^^10^ The effects of subtle genotype-dependent gene expression may impact microglia functions and ultimately disease risk over the course of a lifetime.

The goal of our study was to identify PD-associated SNPs that potentially function via allele-dependent regulation of gene expression in microglia. We first mapped open chromatin in induced pluripotent stem cell (iPSC)-derived microglia using assay for transposase accessible chromatin with sequencing (ATAC-seq). We combined our data with published ATAC-seq data from primary microglia^11^ to create a consensus list of open chromatin regions. We then overlapped these consensus regions with published H3K27ac chromatin immunoprecipitation sequencing (ChIP-seq) datasets from primary *ex vivo* microglia tissue^11^ to demarcate active enhancers and promoters. These regions overlapped 73 out of 6,749 “SNPs of interest” published in the latest GWAS metanalysis.^1^ We report these as candidates for in-depth mechanistic evaluation in microglia.^12^

For thorough functional analysis, we chose to focus on one of our top candidate regulatory elements, an intragenic “risk enhancer” at *SNCA*, defined by its overlap with a PD risk SNP. GWASs of PD have uncovered many risk variants at the *SNCA* locus which make up at least three independent association signals.^13^^,^^14^ However, distinct functional variants at this locus have yet to be identified in microglia. In this study we report on two variants, rs2737004 and rs2619356, that we believe to be functional in microglia. They are in linkage disequilibrium (LD) with a PD-association signal spanning the 5′ end of *SNCA* into *MMRN1*. CRISPR/Cas9 deletion of the open chromatin region, containing these variants, led to reduced expression of *SNCA* and the adjacent gene *MMRN1*, confirming a regulatory effect on nearby genes. In addition, there was a small subset of differentially expressed genes involved in cell-cycle regulation and glucose metabolism, which are two linked processes involved in microglia inflammatory responses. Our work underscores the importance of evaluating genetic risk on a cell-type-specific basis and implicates *SNCA*, and a specific association signal there, as an important risk locus in microglia.

## Results

### Regions of accessible chromatin are consistent between iPSC-derived and primary microglia

We evaluated genetic risk in microglia using the iPSC-derived microglia model developed by McQuade et al.^15^ Following the differentiation of iPSCs to mature microglia, we performed ATAC-seq to map genome-wide regions of accessible chromatin (n = 4). iPSC-derived model systems represent a promising alternative to primary tissue due to their relative ease of creation and experimental manipulation as well as the ability to follow their trajectory through differentiation. However, culturing cells in an *in vitro* environment may affect the chromatin landscape.^11^ Therefore, to augment our newly generated ATAC-seq data from iPSC-derived microglia, we reanalyzed ATAC-seq data from 13 *ex vivo* primary microglia samples published by the Glass laboratory.^11^ For these samples, microglia were isolated from the brain tissue of 13 different patients, both male and female, ranging in age from 2 to 17 years. The samples were derived from various regions of the brain, including the temporal cortex, the frontal cortex, the occipital cortex, and the cerebellum. We combined the datasets to identify active chromatin regions in iPSC-derived microglia shared with a heterogeneous population of primary microglia. Presumably, these common loci are relevant to a broad range of microglia types.

Many ATAC peaks are shared between iPSC-derived microglia and primary microglia. Out of 73,276 peaks present in iPSC-derived microglia, 60,139 (∼82%) overlap with primary microglia (Figure S1A). An example of peak consistency at a particular locus is shown in Figure S1B around *CX3CR1*, a gene known to be expressed in microglia. For subsequent data analyses, we used regions of open chromatin that were present in both iPSC-derived and primary microglia.

### 73 out of 6,749 PD risk SNPs overlap functional regulatory DNA in microglia

SNPs that reside in active regulatory DNA are known to function by disrupting transcription factor binding, leading to changes in gene expression.^16^ To identify such SNPs, we first overlaid the location of PD risk "SNPs of interest" from Nalls et al.^17^ with consensus iPSC-derived and primary microglia ATAC-seq peaks. We also combined that data with published H3K27ac ChIP-seq data from 3 primary ex *vivo* microglia samples^11^ to narrow down ATAC-seq peaks within regulatory DNA. Out of 6,749 SNPs, 73 were in ATAC-seq peaks and were in or within 100 bp of an H3K27ac ChIP-seq peak (Table S1).

From the 73 SNPs in ATAC peaks, we highlight six top candidate risk SNPs (Table 1, and related Table S1) ranked by GWAS p value. Other than rs4889599, we observed that these SNPs are in ATAC peaks that are flanked by H3K27ac ChIP-seq peaks (Figure S2). This pattern is what we typically consider optimal when evaluating candidate loci because it displays nucleosome displacement surrounded by acetylated histone marks, indicative of transcriptional activity and transcription factor occupancy. Indeed, a MotifbreakR analysis showed that 51 out of the 73 SNPs in ATAC-seq peaks are predicted to have an allele-specific preference for one or more transcription factors expressed in microglia (Table S2).^18^

**Table 1:** Top candidate risk SNPs that overlap active regulatory DNA

SNP	Closest gene	GWAS p value	Function of gene	Role in neurodegeneration
rs12726330	*SLC50A1* (SWEET1)	2.208x10^−27^	glucose transport across the cell membrane^45^	No studies have directly evaluated SLC50A1, but decreased expression of glucose transporters correlates with hypometabolism of glucose in neurodegenerative diseases.^46^
rs2737004	*SNCA*	7.60x10^−11^	regulation of dopamine release, induces fibrilization of microtubule-associated protein tau, and protection against apoptosis by inhibiting p53 and caspase-3 activation^47^	It is mostly known to form toxic aggregates in neurons, but, in iPSC-derived macrophages from patients with an *SNCA* triplication, phagocytosis and cytokine release was impaired.^48^^,^^49^
rs144814361	*BAG3*	9.07x10^−11^	co-chaperone that interacts with Hsp70 to prevent apoptosis during aging and under conditions of stress^49^	The protein is protective against neurodegeneration by preventing NLRP3 activation and inflammation in microglia.^50^
rs3813020	*FBXL19*	2.05x10^−10^	regulates the ubiquitination and degradation of inflammatory cytokines,^51^ regulates RhoA signaling^52^	Its role in neurodegeneration has not directly been tested. However, loss of RhoA is associated with microglia dysfunction and neurodegeneration.^53^
rs4889599	*SETD1A*	7.34x10^−10^	histone methyltransferase that regulates transcriptional programming during embryogenesis^54^	Loss of function is associated with neurodevelopment disorders and dysfunctional neuronal metabolism.^55^ It is a GWAS-identified PD risk gene, but its functional involvement in the disease is unknown.^56^
rs823114 rs7536483	NUCKS1	4.35x10^−9^	involved in cell growth, proliferation, DNA repair, metabolism, and inflammatory and immune responses^57^	The gene is part of the PARK16 locus (one of the first to be identified by PD GWAS). Its expression is downregulated in PD patients, but its causal mechanisms are unknown.^58^

Table of top-ranking SNPs (based on GWAS p value) in open chromatin at active regulatory regions of DNA in microglia. See also Table S1 and Figure S2.

### A “risk haplotype” resides in an intragenic *SNCA* enhancer

**Figure 1:**
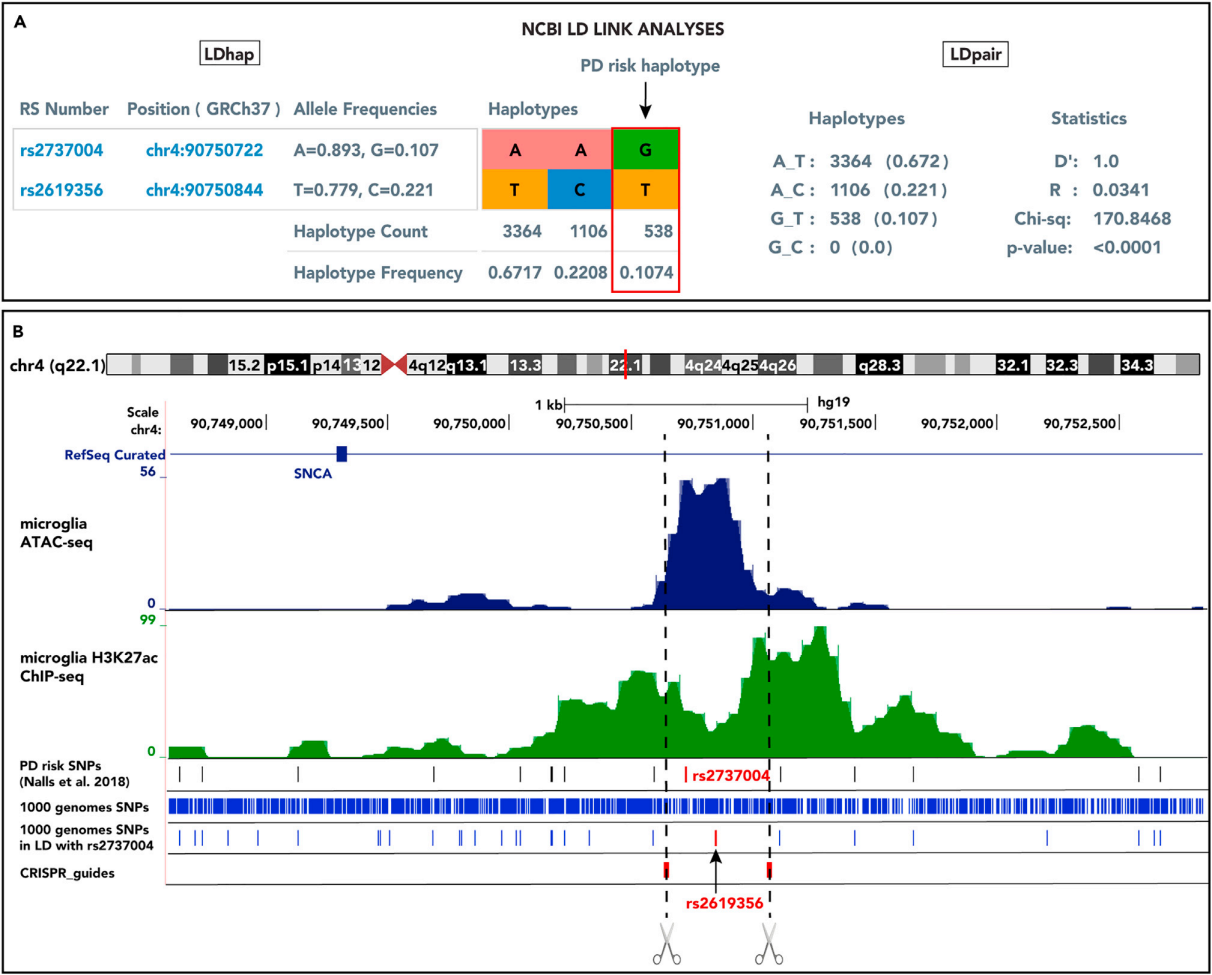
A “risk haplotype” resides in an intragenic *SNCA* enhancer (A) Correlation analysis of the alleles of rs2737004 and rs2619356 using NCBI’s LDlink tools. The LDhap results show the frequency of each allele of the SNPs individually in all populations from NCBI. The colored boxes represent the haplotypes observed in the same population, with their counts and frequencies displayed below. The LDpair analysis reports the calculated statistics for linkage disequilibrium (D′ and R^2^) and the “goodness-of-fit” (chi-square and p value), which indicates the degree that the observed haplotype frequencies deviate from the expected allele frequencies. (B) Genome browser view of microglia ATAC- and H3K27ac ChIP-seq signals plotted with the locations of PD risk SNPs, all SNPs from 1,000 genomes, and the SNPs that are in LD with rs2737004. Rs2619356 was the only other SNP to overlap the ATAC-seq peak. Dotted lines represent the location of CRISPR/Cas9 guides designed to delete the 439 base pair region encompassing both SNPs in the “risk haplotype.”.

*SNCA* is a well-known PD risk gene that has multiple PD GWAS association signals at its locus, yet there are virtually no studies that have dissected genetic risk in microglia at this region. As an in-depth follow-up we focused on an enhancer in intron two of *SNCA* that overlaps one of our top-ranking candidate risk SNP, rs2737004. Our initial goal was to zoom in on this region to determine if there are other SNPs that are in LD with rs2737004. NCBI’s LDproxy tool shows one additional SNP (rs2619356) that is in LD with rs2737004 and maps to the open chromatin region of the same *SNCA* enhancer (Figure 1B). The LDpair tool showed that the two SNPs are in LD (D' = 1), where the G allele of rs2737004 always co-segregates with the T allele of rs2619356 (Figure 1A). Herein we refer to this allele pair as the "risk haplotype". Note that the R^2^ value is low because the allele frequencies in each haplotype are not equal (i.e., one allele is less common than the other). Our previous analysis showed that SNPs with unequal allele frequencies tend to have larger effects.^12^ In this case the minor allele frequency of rs2737004 is not proportionate to the major allele (G = 10.7% and A = 89.3% in all NCBI populations). Furthermore, the G allele of rs2737004 is only present with the T allele of rs26191356 in a smaller subset of all populations (10.74%). We therefore predict that the risk haplotype could have relatively large effects, diluted when large populations are analyzed. It is important to note that rs2737004 is not near the most significant GWAS-associated signal tagged by rs356182 (p value = 1.85 x 10^82^). The two SNPs are also not strongly correlated (D' = 0.6786, R^2^ = 0.0816). Instead, rs2737004 appears to be in LD with a different independent signal tagged by rs763443 (D' = 0.9484) that is located at the opposite end of the gene. These risk signals are reviewed elsewhere by Pihlstrom et al.^14^

### The *SNCA* risk enhancer shows evidence of functionality via 3D chromatin interactions and transcription factor binding

**Figure 2:**
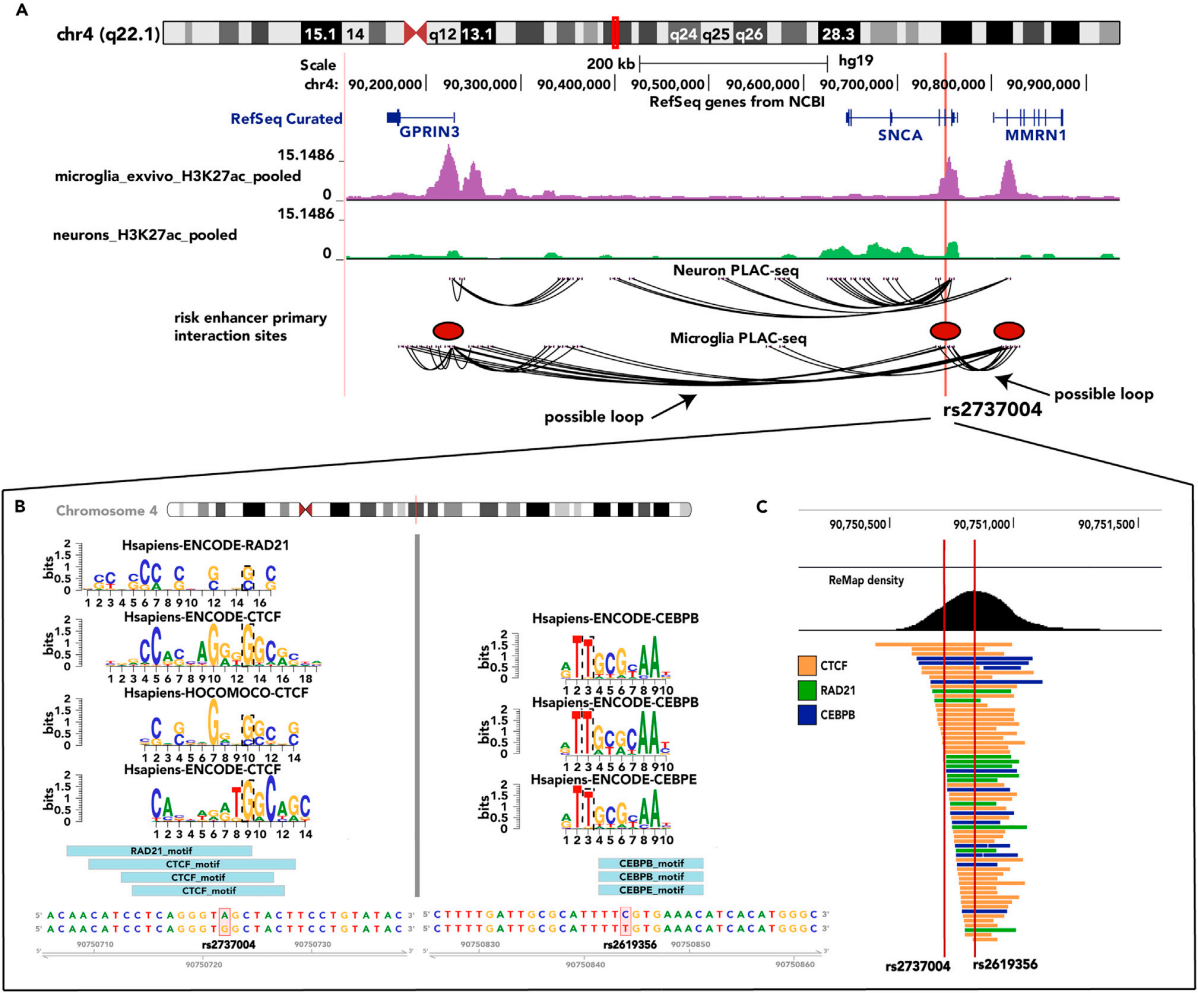
The *SNCA* risk enhancer shows evidence of functionality via 3D chromatin interactions and transcription factor binding (A) H3K27ac ChIP-seq tracks are displayed for microglia (purple) and neurons (green). Below are tracks showing PLAC-seq data for the same cell types. The red ovals denote primary interaction sites, in microglia, of the risk enhancer where rs2727004 is located. (B) MotifbreakR results showing transcription factor (TF) binding motifs of the TFs that have preference for the alleles of the risk haplotype (G for rs2737004 (left) and T for rs2619356 (right)). The letter size represents the results of the positional weight matrix that measures the frequency that the transcription factor binds to that nucleotide. In that same plot, the dashed black box demarcates the position of the SNP. The light blue boxes below represent the positions of the transcription factor binding motifs relative to the SNP’s genomic position, demarcated with the red box. (C) Remap ChIP-seq data for the transcription factors displayed in part B. The red lines show the position of each SNP within the ChIP-seq peak.

Judging by proximity, *SNCA* is the most likely target gene of the intragenic enhancer encompassing rs2737004 and rs2619356. However, enhancers are known to control multiple genes in *cis* and *trans*.^19^ To profile close-range interactions of the risk enhancer, we used published PLAC-seq (proximity ligation-assisted ChIP-seq) data from human microglia, neurons, and oligodendrocytes^20^ (Figure 2A). Compared to neurons and oligodendrocytes, microglia have a unique interaction profile characterized by frequent contact with *MMRN1* and *GPRIN3* (denoted by red ovals in Figure 2A). In microglia, most of the contact points correspond with the presence of an H3K27ac signal, indicating that these 3D interactions happen between the portion of *SNCA* that contains the risk enhancer and active regulatory DNA at *MMRN1* and *GPRIN3*. The interaction profile is different in neurons, where some of the contact points are present within the H3K27ac signal. However, the interactions appear to be more localized around *SNCA* and the intergenic region between *SNCA* and *GPRIN1*. In oligodendrocytes there are few 3D interactions, all of which are between *SNCA* and *GPRIN1* (data not shown). This suggests that the *SNCA* risk enhancer functions differently in microglia than in neurons or oligodendrocytes and highlights the cell-specific activity of the risk enhancer.

Both SNPs of the "risk haplotype" could have a synergistic influence on gene expression levels of *SNCA* or other genes via allele-specific binding to transcription factors. As a first approximation for such a mechanism, we used MotifbreakR^18^ to find transcription factors that are predicted to have differences in binding strength to the protective and risk alleles of rs2737004 and rs2619356. The most frequent motif altered by rs2737004 genotype was CTCF, which shows a preference for G (the risk allele) (Figure 2B; Table S2). RAD21, a core subunit of the cohesin complex, also shows a preference for the G allele (Figure 2B; Table S2). To provide supporting evidence of CTCF and RAD21 binding at rs2737004, we searched ChIP-seq data from ReMap.^21^ Although microglia are not a cell type in the ReMap database, multiple other cell types, including peripheral macrophages (a related cell type), show binding of CTCF at rs2737004 (Figure 2C orange bars). There is also evidence of RAD21 binding at the same location (Figure 2C blue bars). CTCF and RAD21 work together to mediate 3D chromatin structure, forming loops between enhancers and promoters to facilitate chromatin accessibility and gene expression.^22^^,^^23^ A plausible mechanism leading to increased risk for PD could thus involve changes in 3D chromatin organization that impact the expression of multiple genes.

The MotifbreakR analyses also showed that rs2619356 influences the affinity for many transcription factors (Table S2). Of the transcription factors that have a strong preference for the T-allele (part of the risk haplotype), CEBP proteins, specifically CEBPB and CEBPE, appeared most frequently (3 motifs) (Figure 2B; Table S2). There was no ReMap data for CEBPE. However, CEBPE binds to a motif at rs2619356 in multiple cell types, including macrophages. CEBPB is a basic-leucine zipper transcription factor that regulates pro-inflammatory responses in microglia.^24^ It has also been shown to bind at *SNCA* and promote its expression in a neuroblastoma cell line.^25^ Others have linked *SNCA* dysregulation to allele-specific transcription factor occupancy.^3^^,^^26^ It is hypothesized that this mechanism leads to subtle increases in *SNCA* expression over a long period of time, contributing to alpha-synuclein aggregation later in life. These data point to a similar mechanism. However, this analysis is a preliminary step in demonstrating the functionality of rs2619356 and rs2737004. Moving forward we focused on identifying the target genes of the risk enhancer created by CRISPR/Cas9-mediated deletion of the open chromatin region of the risk enhancer.

### The *SNCA* risk enhancer controls expression of *SNCA*, *MMRN1*, and a network of additional genes in microglia

When defining risk genes, a common approach is to implicate the nearest genes to the SNP. Whereas this approach has provided substantial insight into PD-related pathways, it may not reveal the full extent of gene targets because an enhancer may affect genes multiple kilobases away on linear DNA or even on different chromosomes.^27^ To determine the target genes of the enhancer in which the risk haplotype resides, we created a 439 bp deletion of the open chromatin region containing rs2737004 and rs26191356 (Figure 1B). We then performed bulk RNA sequencing (RNA-seq) at three time points across differentiation of microglia: day 0 iPSCs, day 12 hematopoietic progenitors (HPCs), and day 40 microglia. Sample names, their collection times, and total number of technical and biological replicates can be found in Figure S4B. There were no significant differences between standard microglia marker genes *AIF1* (*IBA1*), *TMEM119*, *CD11b*, and *P2RY12* in wild-type compared to edited cell lines (Figure S5C). Note that *TMEM119* mRNA expression was low in microglia. The use of this marker as a robust indication of microglia has been recently challenged due to its variability, both increased and decreased, depending on activation status.^28^ We also confirmed protein expression by immunocytochemistry (ICC) of TMEM119 and IBA1 in all cell lines (Figure S5B). We did not do a formal quantification due to inconsistencies related to timing and cell losses during the staining procedure.

**Figure 3:**
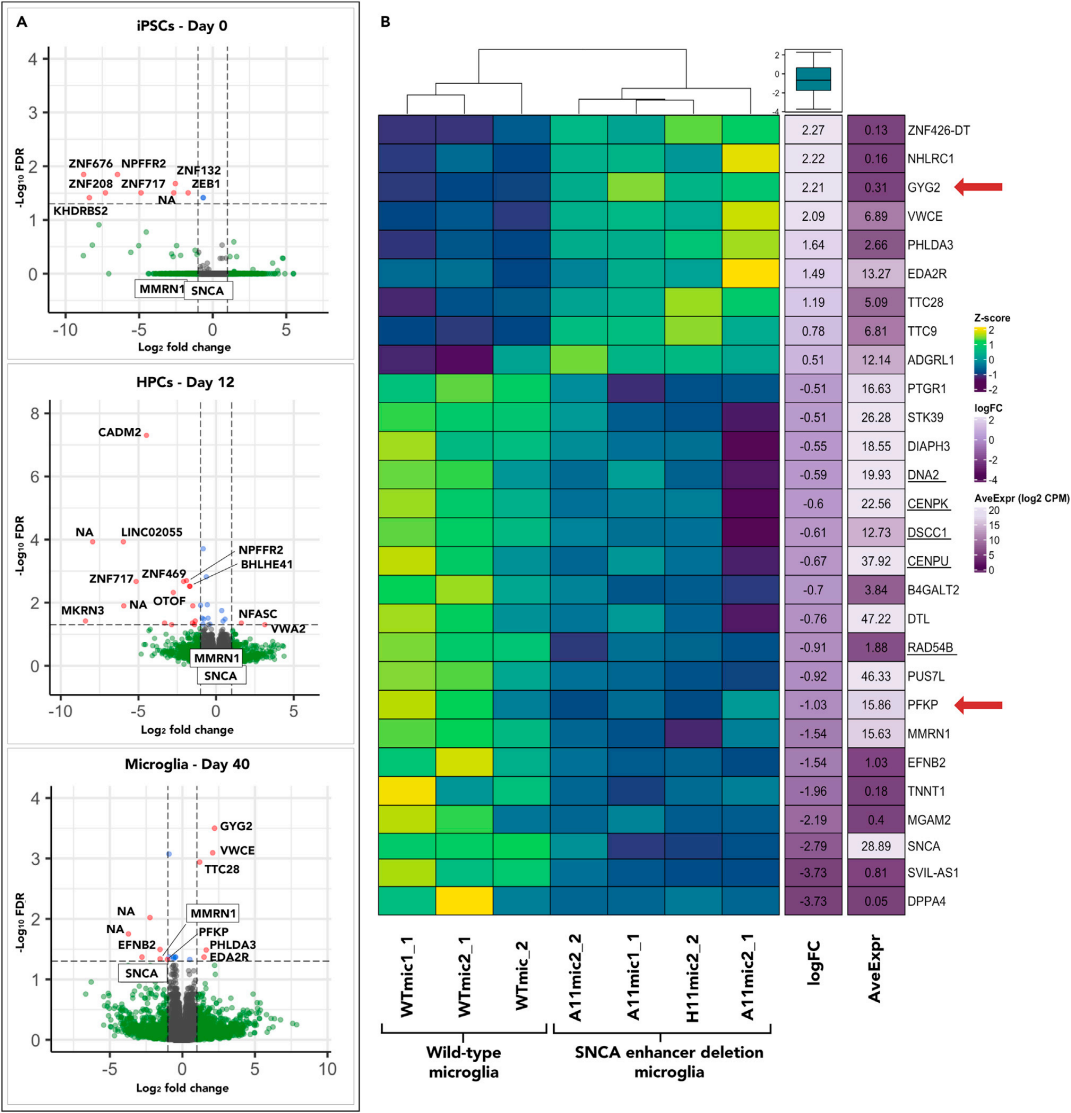
The SNCA risk enhancer controls the expression of *SNCA*, *MMRN1*, and a network of additional genes in microglia (A) EdgeR glmFit results comparing wild-type to *SNCA*-deletion cell lines across three time points in differentiation. Dotted lines represent a Log2 fold change of 2 and a false discover rate (FDR (Benjamini-Hochberg)) of 0.05. Gene name labels were manually added for consistency and clarity. See also Table S3 for results. (B). Heatmap of *Z* scores calculated from TMM-normalized log2 counts per million (log2 CPM). The genes in the heatmap represent those that have a Benjamini-Hochberg FDR cutoff of less than 0.1 (less stringent than used for the volcano plots in part A), and a fold change (FC) of over 1.4 (log FC > 0.5). The final plot was made by ranking the genes by log FC and taking the 15 genes at the top and bottom of that list.

Looking across time points, we observed that the number of genes influenced by the enhancer deletion increased with the differentiation time course (Figure 3A and related Table S1). There was also little overlap between differentially expressed genes at each stage, suggesting that either the enhancer is not as active at earlier time points or it controls different sets of genes at each stage in differentiation. Interestingly, *SNCA* and *MMRN1* expression significantly decreased in microglia but was unchanged in iPSCs and HPCs (Figure 3A), indicating a unique role for the risk enhancer in controlling expression of *SNCA* and *MMRN1* in microglia. Our findings also corroborate the PLAC-seq data showing that *MMRN1*, in addition to *SNCA*, is a likely target of the risk enhancer in microglia. The PLAC-seq data indicated that *GPRIN1* could be a target gene. However, its expression was not significantly different at any time points after the enhancer deletion.

Genes with a differential expression false discovery rate (FDR) of ≤0.1 in iPSCs and HPCs showed no enrichment of pathways identified by GOnet gene ontology (GO) analysis tool.^29^ Using the same cutoff in microglia, the only pathway to show enrichment was DNA conformation change (FDR = 1.2e-2), which is described by the Gene Ontology Resource as “a cellular process that results in a change in the spatial configuration of a DNA molecule.” A conformational change can bend DNA or alter the twist, writhe, or linking number of a DNA molecule.^30^” Seven out of the 42 genes, *CENPK*, *DNA2*, *HELLS*, *DSCC1*, *CENPU*, *RAD54B*, and *MCM8*, were a part of this network. The genes that had an FDR value ≤0.05 are displayed and underlined on the heatmap in Figure 3B. Looking more specifically, they are known to be involved in DNA replication and are likely indicative of changes in the cell cycle. This was confirmed using GO enrichment from the Gene Ontology Resource (FDR = 2.66E-03).^30^ We also observed an inverse relationship between *GYG2* (Glycogenin 2) and *PFKP* (Phosphofructokinase) expression (Figure 3B red arrows). *GYG2*, a gene involved in glycogen biosynthesis, was most significantly upregulated while *PFKP*, a gene involved with the conversion of fructose 6-phosphate to fructose 1,6-bisphosphate at the beginning stages of glycolysis, was downregulated. This, along with changes in cell-cycle genes, suggests that loss of the enhancer could be affecting glucose metabolism and possibly shifting glucose utilization to glycogen storage rather than glycolysis. This is a phenotype that we plan to explore in future experiments.

## Discussion

Here we aimed to determine a subset of PD-associated risk SNPs located in regions of active regulatory DNA in microglia and to identify functional risk SNPs in this cell type. In doing so, we substantially narrowed down the 6,749 PD-associated "SNPs of interest" from the latest PD metanalysis to a more tractable list of 73. We chose one candidate risk locus, *SNCA*, for more in-depth evaluation based on its overlap with a top-ranking candidate SNP, rs2737004 (GWAS p value = 7.6x10ˆ-11, OR = 1.14), in addition to the relevance of *SNCA* to PD risk, which is not well understood in microglia.

Multiple PD GWASs have reported independent association signals at the *SNCA* locus, but it is still unclear which variants are functional around these signals.^1^^,^^14^^,^^31^ The top-ranking GWAS hit, rs356182 (p value = 1.85 x 10^−82^), is located at the 3′ end of *SNCA* in a regulatory element identified in neurons.^26^ Our lab previously demonstrated that heterozygous deletion of rs356182 causes changes in the expression of *SNCA* and thousands of additional genes, many of which are related to neuronal differentiation.^26^ However, that same 3′ enhancer is not active in microglia and none of the risk variants near rs356182 overlap microglia H3K27ac and ATAC signals. Alternatively, our analysis points to a potential functional SNP in microglia, rs2737004, that appears to segregate independently of rs356182 (D’ = 0.6786, R^2^ = 0.0816). Rs2737004 is linked to a separate independent GWAS signal, near the 5′ end of *SNCA*.^13^^,^^14^ It is in LD with the top tag SNP rs763443 (D’ = 0.9484). We speculate that the risk signal tagged by rs763443 represents a group of functional variants in microglia (or other immune cells), and our analysis pinpoints rs2737004 as a top functional candidate. In contrast, the risk signal at rs356182 may represent functional variants in other cells such as neurons. In the larger context of PD, we imagine a scenario where multiple nearby genetic risk signals each function through *SNCA*, but these separate signals represent biology relevant to different cell types. Thus, each set of regulated genes is unique, resulting in dysregulation of different cellular processes. For these reasons, we believe that, even though rs356182 carries the strongest association with PD risk, it is not the most relevant candidate for follow-up in microglia and more focus should be placed on understanding the function of PD-associated variation at the independent signal at the 5′ end of *SNCA*.

Upon further evaluation of the *SNCA* locus, we located an additional SNP, rs2619356, in the risk enhancer that is in strong LD (D’ = 1) with rs2737004. Interestingly, one version of the haplotype is non-existent in the study population and the risk allele of rs2737004 only appears with the T allele of rs2619356, indicating co-segregation/linkage of the two alleles. Rs2619356 was not published in the original list of “SNPs of interest” likely due to the use of R^2^ as a measure of LD, which results in the exclusion of SNPs with allele frequencies that are not close to 50%. In terms of our approach to include functional SNPs based on their location in open chromatin at active regulatory DNA, rs2619356 is positioned in the center of the open chromatin region and transcription factor binding ReMap signal (Figure 2C). It is also predicted to have allele-specific preference for multiple transcription factors (Table S2), making it an equally relevant candidate for mechanistic follow-up.

A plausible mechanism of the risk enhancer could involve alterations in chromatin looping mediated by allele-specific CTCF and RAD21 binding. The G allele of rs2737004 has a stronger preference for CTCF and RAD21, which could promote the stability of the loop to facilitate *SNCA* and *MMRN1* expression. In 3 out of the 4 tissues profiled (breast, esophagus, and pituitary) by the Genotype-Tissue Expression project (GTEx), the GG genotype of rs2737004 is associated with increased expression of *SNCA*. The presence of CEBP within the motif for rs2619356 and its preference for the allele of the risk haplotype supports this idea, as CEBP has also been shown to promote the expression of *SNCA*. Studies, primarily in neurons, demonstrate that elevated expression of *SNCA* leads to alpha-synuclein aggregation and cellular dysfunction. However, the function of endogenous *SNCA* in microglia is not well known. *SNCA* has multiple roles that could require time-specific or varying levels of *SNCA* expression in different cell types. For example, alpha-synuclein is highly expressed in neurons and required for many important neuronal functions such as differentiation and synaptic transmission.^32^ Alpha-synuclein has more recently been shown to facilitate immune responses and thus may be lowly expressed under homeostatic conditions in immune cells like microglia but becomes upregulated on a temporal basis in response to stress or infection.^33^^,^^34^ In different cellular contexts, *SNCA* may therefore be regulated differently via cell-type-specific enhancers. This could explain the differences we observed in topology and regulatory signals like H3K27ac at *SNCA* when comparing microglia to neurons and provides a hypothetical scenario for how PD-associated variants within regulatory elements increase disease risk in distinct cell populations. However, this has not been directly demonstrated and how *SNCA* regulation is carried out in different cell types and different contexts such as viral infection and stress is still an early area of research.

Enhancer looping to target promoters is one of the critical aspects of proper gene regulation. Although the resolution of PLAC-seq data is not precise enough to distinguish whether the interaction of *MMRN1* is with the risk enhancer, the promoter of *SNCA*, or both, there does appear to be evidence for loop formation between *SNCA* and *MMRN1* (Figure 2A). This points to *MMRN1* as a potential target gene. We further believe that *MMRN1* is a plausible target, as deletion of the enhancer led to loss of *MMRN1* expression in addition to reduced *SNCA* expression. Whether the expression changes in the remaining set of genes are due to alterations in primary enhancer interactions or secondary downstream effects is still an open question.

*SNCA* expression changes, both up and down, have been associated with PD risk, but fewer studies have focused on how *MMRN1* relates to PD risk. *MMRN1* is mostly known to function in platelets to aid in coagulation, but there is limited understanding of its physiological functions.^35^ Although the gene has been mostly studied in the context of cancer metastasis, a transcriptome-wide association analysis recently identified *MMRN1* as a gene whose expression associates with PD risk.^36^ *MMRN1* genetic abnormalities have also been found in autosomal dominant PD.^37^ Surprisingly, *SNCA* and *MMRN1* expression was not significantly different in iPSCs or HPCs, whereas in microglia, deletion of the enhancer led to a loss in their expression. We hypothesize that the enhancer promotes or maintains *SNCA* and *MMRN1* expression as the cells differentiate into microglia. This suggests a biologically important role for both *SNCA* and *MMRN1* in microglia, but more studies are needed to corroborate this hypothesis and to understand their function in PD.

To evaluate whether our differentially expressed gene set contained any genes that are known to be dysregulated in PD patients, we looked for overlap with published gene sets from cells with genetic abnormalities in *SNCA*.^38^ We confirmed overlap of 5 genes in our dataset with a gene set from A53T *SNCA* mutant dopaminergic neurons. In addition to *SNCA*, *MMRN1*, *DTL*, and *DPPA4* were upregulated whereas *PFKP* was downregulated in comparison to wild-type control cells. In a PD patient-derived dopaminergic cell line with an *SNCA* triplication, *SNCA* was upregulated, but *VWCE*, *EDA2R*, *PUS7L*, and *MGAM2* were downregulated. This suggests that changes in *SNCA* may affect a common set of genes in dopaminergic neurons and microglia. However, the changes we observed in our model may not be completely related to *SNCA* expression changes but more related to the loss of the enhancer that controls a specific set of genes, including *SNCA*, that are relevant to microglia function.

Two other genes stood out due to their involvement in glucose metabolism, which is a key process in microglia that controls their activation in response to inflammation.^39^ The most upregulated gene, *GYG2*, is involved in glycogen biosynthesis. We also observed a loss of *PFKP*, which is a critical regulatory enzyme in glycolysis.^40^ Our GO analysis identifying cell cycle as a process affected by 7 of the other differentially expressed genes supports a role for this metabolic phenotype in PD risk as metabolism and cell cycle are highly interconnected.^41^ On a related note, out of the 73 SNPs in microglia regulatory DNA, the one with the most significant GWAS p value is at *SLC50A1* (Table 1; Figure S2), a glucose transporter whose function has never been studied in microglia. There have been clinical findings showing that PD patients have reduced glucose metabolism at early stages of the disease.^42^ Strikingly, altered glucose metabolism in diabetes patients was also found to increase the chances for developing PD by 30%.^43^ Our results and the findings of others justify the need for more studies to understand how dysfunctional glucose metabolism in microglia leads to increased risk for PD as this pathway may be a promising therapeutic target in PD.

To the best of our knowledge this is the first functional evaluation of a “risk enhancer” near the PD-association signal at the 5′ end of *SNCA*. How *SNCA*, *MMRN1*, and other genes are regulated by this enhancer may play an important part in PD pathogenesis by impacting inflammatory functions in microglia. Our data also provide a starting point for dissecting genetic risk at other loci and demonstrate the importance of careful evaluation of PD-associated variants on a cell-type-specific basis. We advocate for more post-GWAS testing of these risk variants to make sense of the genetic contribution to increased PD risk. There are currently no treatments to modify the progression of PD. Additional studies that build on our findings will help understand the complex genetic etiology of PD and identify alternative disease-modifying targets.

### Limitations of the study

Although we demonstrated a role for the risk enhancer in controlling a network of genes, the mechanisms of the SNPs within enhancers remain to be determined. We attempted to create single-base-pair edits to generate isogenic cell lines with different genotypes of rs2737004. However, due to evidence of confounding effects on gene expression (possibly off-target edits), we did not move forward with that portion of the study. This limitation is worth noting because of the challenges related to technical constraints to single-base-pair-editing, requiring a PAM sequence near the targeted nucleotide. The choice of guide sequences is much more restricted which increases the chances for off-target edits. Future follow-up studies using cell lines with germline variation, similar to the methods of Langston et al.,^44^ may be a better approach.

## STAR★Methods

### Key resources table

**Table:** 

REAGENT or RESOURCE	SOURCE	IDENTIFIER
**Antibodies**		

Mouse APC anti-human CD43	BioLegend	cat#343205; RRID: AB_2194072
Mouse anti-human Iba1	Thermo Fisher Scientific	GT10312; cat#MA5-27726; RRID: AB_2735228
Alexa Fluor 488 goat anti-mouse	Thermo Fisher Scientific	cat#A-11001; RRID: AB_2534069
Rabbit anti-human TMEM119	Thermo Fisher Scientific	cat#PA5-119902; RRID: AB_2913474
Alexa Fluor 594 goat anti-rabbit	Thermo Fisher Scientific	cat#A-11012; RRID: AB_2534079

**Chemicals, peptides, and recombinant proteins**		

insulin-transferrin-selenium (100X)	Thermo Fisher Scientific	cat#41400045
B27	Thermo Fisher Scientific	cat#17504044
N2	Thermo Fisher Scientific	cat#17502048
glutamax	Thermo Fisher Scientific	cat#35050061
non-essential amino acids	Thermo Fisher Scientific	cat#11140050
monothioglycerol	Sigma	cat#A8960-5G
human insulin	Sigma	cat#I2643-50MG
IL-34	Peprotech	cat#200-34
TGFβ1	Peprotech	cat#100-21
M-CSF	Peprotech	cat#300-25
CD200	Novoprotein	cat#C311
CX3CL1	Peprotech	cat#300-31
Poly(ethyleneimine) solution (PEI)	Milipore Sigma	cat#181978-100g
4% paraformaldehyde	Electron Microscopy Sciences	cat#157-8-100
DPBS	Gibco	cat#14190-144
Goat serum	Abcam	cat#ab7481
Triton-x	Fisher Scientific	cat#AAA16046AE
NucBlue fixed stain ready probes	Thermo Fisher Scientific	cat#R37606
Poly-D-Lysine	Gibco	cat#A38904-10
Tris-HCl pH 7.5	Thermo Fisher Scientific	cat#15567-027
NaCl	Fisher	cat#S271-500
MgCl_2_	Fisher	cat#bp214-500
nuclease free H_2_O	Invitrogen	cat#AM9938
NP-40	Thermo Fisher Scientific	cat#85124
Tween-20	Fisher	cat#bp337-500
Digitonin	Promega	cat#G9441

**Critical commercial assays**		

hematopoietic kit	STEMdiff	cat#05310
Nextera DNA library prep kit	Illumina	cat#FC-121-1030
Zymo clean and concentrator kit	Zymo	cat# D4014
QuantiFluor® dsDNA System	Promega	cat#E2671
NextSeq 500/550 150 bp sequencing kit (v2)	Illumina	cat# 20024907
Lonza Human Stem Cell Nucleofector Kit 1	Lonza	cat#VPH-5012
QIAshredder	Qiagen	cat#79654
RNeasy isolation kit	Qiagen	cat#74104
Takara SMARTer Stranded Total RNA-Seq Kit v3 Pico Input Mammalian	Takara Bio	cat#634485
QIAprep Spin Miniprep Kit (250)	Qiagen	cat#27106
QIAquick Gel extraction kit	Qiagen	cat# 28704/28706

**Deposited data**		

raw and analyzed data	this paper	GEO: GSE245524
reanalyzed microglia ATAC-seq and ChIP-seq data	Gosselin et al.^11^	dbGAP, accession number: phs001373.v1.p1
reanalyzed PLAC-seq data	Nott et al.^20^	dbGAP, accession number: phs001373.v2.p1

**Experimental models: Cell lines**		

Human iPSCs	ATCC-DYS0100	cat#ACS-1019; RRID:CVCL_X499
Human iPSCs	Synthego	PGP1-SV1

**Oligonucleotides**		

Illumina primer 1 AATGATACGGCGACCACCGA	IDT	N/A
Illumina primer 2 CAAGCAGAAGACGGCATACGA	IDT	N/A
SNCA_guide_1_F caccgGTGAAGGTATCCGTATAATG	this paper	N/A
SNCA_guide_1_R aaacCATTATACGGATACCTTCACc	this paper	N/A
SNCA_guide_2_F caccgCAATGACTTTCGGTATACTG	this paper	N/A
SNCA_guide_2_R aaacCAGTATAccGAAAGTCATTGc	this paper	N/A

**Recombinant DNA**		

pSpCas9(BB)-2A-GFP (PX458)	Addgene Ran et al.^72^	cat#48138; RRID:Addgene_48138

**Software and algorithms**		

Illumina NextSeq Control Software (NCS) v2.0	Illumina	https://support.illumina.com/sequencing/sequencing_instruments/nextseq-500/downloads.html
Illumina Bcl2fastq v1.9.0	Illumina	https://support.illumina.com/sequencing/sequencing_software/bcl2fastq-conversion-software/downloads.html
Trimgalore	Felix Krueger, Babraham Institute	https://github.com/FelixKrueger/TrimGalore
BWA v0.7.17	Li et al.^60^	https://anaconda.org/bioconda/bwa; RRID:SCR_010910
Multiqc v1.0	Ewels et al.^61^	https://anaconda.org/bioconda/multiqc; RRID:SCR_014982
Samblaster v0.1.24	Faust et al.^62^	https://anaconda.org/bioconda/samblaster; RRID:SCR_000468
Samtools v1.9	Danecek et al.^73^	https://anaconda.org/bioconda/samtools, RRID:SCR_002105
MACS2 v2.1.1	Zhang et al.^64^	https://anaconda.org/bioconda/macs2; RRID:SCR_013291
GenomicRanges v3.11	Lawrence et al.^65^	https://bioconductor.org/packages/release/bioc/html/GenomicRanges.html; RRID:SCR_000025
Bedtools v2.29.0	Quinlan et al.^66^	https://anaconda.org/bioconda/bedtools; RRID:SCR_006646
Benchling	N/A	https://www.benchling.com/RRID:SCR_013955
IGV v2.16.2	Robinson et al.^74^	https://igv.org/doc/desktop/#DownloadPage/; RRID:SCR_011793
ChIPseeker v3.11	Wang et al.^75^ Yu et al.^67^	https://bioconductor.org/packages/release/bioc/html/ChIPseeker.html; RRID:SCR_021322
STAR v2.5.4b	Dobin et al.^70^	https://anaconda.org/bioconda/star;
edgeR v3.18	Robinson et al.^76^	https://bioconductor.org/packages/release/bioc/html/edgeR.html; RRID:SCR_012802
Graphpad Prism v10.0.3	N/A	https://www.graphpad.com; RRID:SCR_002798
UCSC Genome Browser PLAC-seq, H3K27ac ChIP-seq, and ATAC-seq session	Nott et al.^20^	https://genome.ucsc.edu/s/nottalexi/glassLab_BrainCellTypes_hg19
UCSC Genome Browser	Nassar et al.^77^	https://genome.ucsc.edu; RRID:SCR_005780

**Other**		

StemFlex medium	ThermoFisher	cat#A3349401
Geltrex LDEV-free reduced growth factor basement membrane	ThermoFisher	cat#A1413201
iMatrix	Matrixome	cat#892012
ReLeSR	STEMCELL Technologies	cat#05872
Cell Staining Buffer	BioLegend	cat#420201
TruStain FcX (Fc Receptor Blocking Solution)	BioLegend	cat#422301
12x75mm round bottom tubes	Fisherbrand	cat#14-965-3C
DMEM/F12, HEPES, no phenol red	ThermoFisher	cat#11039021
8-well chamber slides	Ibidi	cat#80841
EverBright Hardset Mounting Medium	Biotium	cat#23003
TD Buffer (part of kit)	Illumina	cat#FC-121-1030
ATM (part of kit)	Illumina	cat#FC-121-1030
NT buffer (part of kit)	Illumina	cat#FC-121-1030
Illumina Nextera DNA unique Dual Indexes	Illumina	cat#20027214
NEBNext High-Fidelity 2X PCR Master Mix	NEB	cat#M0541L
KAPA Pure beads	Roche	cat#KK8001
Agilent DNA High Sensitivity chip	Agilent Technologies	cat#5067-4626
T4 Kinase (PNK)	Thermo Fisher Scientific	cat#EK3001
BbsI	NEB	cat#R0539S/R0539L
Cut Smart Buffer	NEB	cat#B7204
T4 ligase buffer	Thermo Fisher Scientific	cat#B69
T4 DNA ligase	Thermo Fisher Scientific	cat# EL0011
PlasmidSafe ATP-dependent DNase	Lucigen	cat#E3101K
PlasmidSafe buffer	Lucigen	not available
25 mM ATP solution	Lucigen	not available
One Shot Top10 E. Coli	Thermo Fisher Scientific	cat#C404003
S.O.C.	Thermo Fisher Scientific	cat#15544034
Revitacell	Thermo Fisher Scientific	cat#A2644501

### Resource availability

#### Lead contact

Further information and requests for resources and reagents should be directed to and will be fulfilled by the lead contact, Alix Booms (alix.booms@vai.org).

#### Materials availability

There are restrictions to the availability of edited PGP1 cell lines due to the requirement of a materials transfer agreement (MTA).

#### Data and code availability

• Bulk RNA-seq and ATAC-seq data have been deposited at GEO and are publicly available as of the date of publication. This paper also analyzes existing publicly available data. Accession numbers are listed in the key resources table.
• This paper does not report original code.
• Any additional information required to reanalyze the data reported in this paper is available from the lead contact upon request.

### Experimental models and study participant details

#### iPSC cell lines

For ATAC-seq experiments, induced pluripotent stem cells (iPSCs) were obtained from ATCC (ACS-1019, DYS0100, male neonate). For CRISPR editing experiments, iPSCs were obtained from Synthego (PGP1-SV1, Male age 55). All validation and QC of cell lines were performed by the supplier. We have not authenticated these cells following receival. Conditions for culturing both cell lines include 5% CO^2^ at 37°C. At the iPSC stage, cells were cultured in StemFlex medium on either Geltrex LDEV-free reduced growth factor basement membrane for the ATCC cell line or iMatrix for the PGP1 cell line. When cells reached 80% confluency, they were passaged using ReLeSR

### Methods details

#### iPSC differentiation to HPCs

iPSCs were first differentiation to hematopoietic progenitors (microglia precursors) using the STEMdiff hematopoietic kit per the methods detailed by McQuade et al.^15^ On day -1 iPSCs are seeded in 6-well plates and allowed to adhere overnight. On day 0, StemFlex is removed and replaced with medium A. On day 2 half of the medium was changed out with fresh medium A. On day 3 all, media was changed to medium B. Half medium changes were then done on days 5, 7, and 10. Cells were harvested on day 12 and assessed for CD43 expression using Flow.

#### Flow analysis of HPC markers

To confirm iPSC differentiation to HPCs on day 12, cells were stained for CD43. We followed the “Cell Surface Flow Cytometry Staining Protocol” from Biolegend. Supernatant and non-adherent cells were collected from each well of a 6-well plate. They were then pelleted and re-suspended in 5 mL for counting. Single suspensions of 200,000 cells were prepared in up to 15 mL of Cell Staining Buffer. Cells were centrifuged at 350 x g for 5 minutes and supernatant was discarded. The pellet was then resuspended in Cell Staining Buffer (100 μl/ # of conditions). Five microliters of TruStain FcX (Fc Receptor Blocking Solution) was added to each sample followed by an incubation at room temperature for 5-10 minutes. After incubation, 200,000 cells per condition were aliquoted into culture test tubes (Fisherbrand 12 × 75mm), one for CD43 and one for the non-stained control. Five microliters of CD43 was then added to one sample and allowed to incubate for 15-20 min in the dark. Cells were washed two times with at least 3 mL of Cell Staining Buffer. At the final wash the pellet was resuspended in 300 ul of Cell Staining Buffer plus 10.9 mM DAPI at a concentration of 3uM. Using a Beckman Coulter CytoFLEX S Flow cytometer. We determined that all cultures were pure if over 90% of cells assayed were positive for CD43 (per McQuade et al.^15^). See Figure S3B and S5 A for Flow results on the ATCC and PGP1 cell lines respectively.

#### Differentiation of CD43^+^ HPCs to microglia

On day 0, HPCs were plated at 100,000 cells per 6-well plate on iMatrix in 2 mL of microglia medium: DMEM/F12, 2X insulin-transferrin-selenium, 2X B27, 0.5X N2, 1X glutamax, 1x non-essential amino acids, 400 μM monothioglycerol, 5 μg/mL insulin. This media was supplemented with 100 ng/mL IL-34, 50 ng/mL TGFβ, and 25 ng/mL M-CSF. On days 2, 4, 6, 8, and 10, 1 mL of microglia medium plus 3 freshly thawed cytokines were added to each well. On day 12, all but 1 mL was collected from each well and spun down at 300 rcf for 5 minutes. The pellet was resuspended in 1mL/well fresh medium plus 3 cytokines and added back to the same plate. Media was supplemented again with 1 mL/ well fresh microglia media plus 3 cytokines on days 14, 16, 18, 20, 22, and 24. On day 25 all but 1 mL was removed from each well and spun down at 300 rcf for 5 minutes. Cells were then resuspended in 1 mL/ well fresh microglia medium plus 5 cytokines (100 ng/mL IL-34, 50 ng/mL TGFβ, 25 ng/mL M-CSF, 100 ng/mL CD200 and 100 ng/mL CX3CL1). Cells were collected on day 28 for ICC and RNA-seq.

#### Immunocytochemistry and imaging microglia

Following differentiation of HPCs to microglia, expression of microglia-specific markers were confirmed using a mouse anti-human Iba1 primary antibody with Alexa Fluor 488 goat anti-mouse secondary antibody. We also used a rabbit anti-human TMEM119 primary antibody with Alexa Fluor 594 goat anti-rabbit secondary antibody.

The staining procedure differed for each cell line. For the ATCC cell line, cells were plated in 24-well plates on glass coverslips coated with PEI and allowed to adhere for 24-hours in the incubator (5% CO^2^ at 37°C). They were then fixed with 4% paraformaldehyde and permeabilized (DPBS, goat serum, and triton-x). Cells were incubated in blocking buffer (DPBS and goat serum) with primary antibodies overnight at 4°C. After 24-hours, cells were washed with DPBS and incubated with secondary antibodies in DPBS for 30 minutes. They were then washed again prior to adding NucBlue Fixed stain ready probes. Coverslips were removed and mounted on glass slides using EverBright Hardset Mounting Medium. For PGP1 cell lines, staining was done the same as the ATCC cell lines, except they were plated on Poly-D-Lysine in Ibidi 8-well chamber slides and allowed to adhere at 37°C for 2 hours prior to fixing. For each cell line imaging was done as follows.

ATCC: Fluorescent images for Figure S5 A were taken with the Nicon Eclipse microscope with NIS Elements software (version 5.11.01) and exported at a tiffs. Bright field images were taken directly in culture plates using the EVOS microscope. The final panel of images were compiled in Power Point.

PGP1 wild-type and edited: Confocal Z-stacks for Figure S5 B were collected using a Zeiss LSM 880 equipped with an Axio Observer 7 inverted microscope body and acquired with Zen Black (version 2.3) software using 405nm diode, 488nm argon ion, and 561nm DPSS laser lines. Emitted light was detected through a Zeiss Plan-apochromat 63x/1.4 NA oil immersion objective, using an Airyscan GaAsP detector. Images were collected sequentially in 1024x1024 pixel resolution, using 0.25um z-steps. Images were acquired with an optical zoom of either 1.0 or 2.0, and individual voxels were therefore 0.13x0.13x0.25um or 0.07x0.07x0.25um (xyz), respectively.

To create Figure S5B, raw czi images were opened in Fiji ImageJ (v1.54f). An average intensity z-projection was generated for each image, inclusive of all channels and z, and saved as a tiff. Any image larger than 1012x1012 pixels were cropped for uniformity. All six average intensity projection images were then concatenated for easier import into the ImageJ plugin QuickFigures^59^ to assemble into the final figure shown.

#### ATAC-seq

The biostatistics core at VARI conducted a power calculation based on ATAC-seq effect size to determine the number of optimal replicates. With three replicates and an average depth of ∼40 reads per million, this study has >80% power to detect, with 95% confidence, peaks with ∼1.8 fold or greater difference in accessibility and ∼99% power for a ∼2-fold difference in accessibility. This calculation was done for the purpose of performing a differential accessibility analysis between wild-type and edited microglia in future experiments. We believe the 4 replicates that we generated in combination with the 13 published ATAC-seq data is sufficient to detect robust peaks in microglia.

Microglia were thawed and cultured for at least one week prior to an ATAC-seq experiment. Samples that yielded the best fragmentation started from a total of 10K, 31K, and 100K cells. The pre-specified number of cells were aliquoted into 1.5 mL tubes and centrifuged at 400 x g for 7 minutes. The supernatant was removed, and the cells were washed once with 50 μl ice-cold PBS. The cells were then resuspended in ice-cold Lysis Buffer containing resuspension buffer (1M Tris-HCl pH 7.5 (final conc. = 10mM), 5 M NaCl (final conc. = 10 mM), 1M MgCl_2_ (final conc. = 3 mM), and nuclease-free H_2_O), 10% NP-40 (final conc. = 0.1% v/v), 10% Tween-20 (final conc. = 0.1% v/v), and 1% Digitonin (final conc. = 0.01% v/v). Cells were then incubated on ice for 3 minutes. One mL of wash buffer (990 μl resuspension buffer + 10 μl Tween-20 (final conc. = 0.01% v/v)) was added to each tube. The tubes were then inverted 3X gently and centrifuged at 500 x g for 5 minutes. For each sample, 10 μl of transposition mix (7.5 μl 2X TD Buffer, 2.05 μl 1X PBS, 0.15 μl 10% Tween-20 (final conc. = 0.1 v/v), 1% Digitonin (final conc. = 0.01% v/v), and 0.15 nuclease-free H_2_O) was added. Five μl of ATM was then added separately to each sample. The samples were incubated for 60 minutes on a thermomixer at 1,000 rpm. Following incubation, the samples were placed on ice, and 5 μl of NT buffer was added to each tube to neutralize the tagmentation reaction. Tubes were then centrifuged at 300 x g at 20°C for 1 minute and incubated at room temp for 5 minutes. DNA purification was done using the Zymo clean and concentrator kit.

For library generation, 5 μl of Illumina Nextera DNA unique Dual Indexes plus 25 μl NEBNext High-Fidelity 2X PCR Master Mix was added to 20 μl of purified transposed DNA. The transposed fragments were amplified starting at 72°C for 5 minutes, 98°C for 30 seconds and then five cycles of 98°C for 10 seconds, 63°C for 30 seconds, and 72°C for 1 minute. qPCR was used to determine how many additional cycles to run on each sample. The PCR mix was composed of 5 μl of the partially amplified library from the previous step, 0.5 μl Illumina primer 1 (25 μM), 0.5 μl Illumina primer 2 (25 μM) 0.75 μl 20X Eva Green, and 5 μl NEBNext High-Fidelity 2X PCR Master Mix. Cycle conditions were set to 98°C for 30 seconds, and 20 cycles of 98°C for 10 seconds, 63°C for 30 seconds, and 72°C for 1 minute. The R vs. cycle number was plotted on a linear scale. Additional cycles were calculated by determining the number of cycles needed to reach 1/3 of the maximum R. PCR was continued on the remaining partially amplified libraries for the appropriate number of cycles calculated in the previous step.

#### Sequencing of ATAC-seq libraries

Library quantification, size selection, and sequencing were carried out by the Genomics Core at VAI. PCR amplified libraries were size selected for fragments 200-800 bp in length using double-sided SPRI selection (0.5x followed by 1x) with KAPA Pure beads. The quality and quantity of the finished libraries were assessed using a combination of Agilent DNA High Sensitivity chip (Agilent Technologies, Inc.), and QuantiFluor® dsDNA System. Seventy five base pair, paired-end sequencing was performed on an Illumina NextSeq 500 sequencer using a 150 bp sequencing kit (v2) to produce a minimum of 50M paired-reads per library. Base-calling was done by Illumina NextSeq Control Software (NCS) v2.0, and the output of NCS was demultiplexed and converted to FastQ format with Illumina Bcl2fastq v1.9.0.

#### Identification of ATAC-seq peaks

Four replicates of one iPSC-derived microglia cell line and thirteen replicates of ATAC-seq data from different primary microglia samples (published data) were used to find consensus ATAC-seq peaks. ATAC-seq peak data from primary microglia were obtained from dbGAP deposited by the Glass lab (see key resources table for accession number).^11^ All data from iPSC-derived and primary microglia were processed in the same way. The sequencing depth for iPSC-derived microglia was about 40-50 million, and the read length was about 75 base pairs. For primary microglia, samples were sequenced to a depth ranging from 20-50 million reads, and the read length ranged from 47-76 base pairs.^11^ Using Trimgalore, reads were trimmed or removed if they were below 20 base pairs in length or had a quality score below 20. All other parameters were default. Forward and reverse reads for iPSC-derived microglia, and single-end reads for primary microglia were then aligned to the hg19 genome using the default settings for BWA v0.7.17.^60^ Multiqc v1.0^61^ was then run on all samples following alignment. Samblaster v0.1.24^62^ was used to sort and mark duplicate reads in bam files, and Samtools v1.9^63^ was used to remove duplicate reads and index the bam files. Peaks were then called using MACS2 v2.1.1^64^ default parameters. GenomicRanges v3.11^65^ was used to generate a consensus peak set (starting from narrowPeak files). We used peaks present in 3/4 samples for iPSC-derived microglia and peaks that were present in 10/13 primary microglia samples.

#### PD risk SNPs ATAC-seq peak intersect

The list of 6,749 SNPs was obtained from the Nalls et al. bioRxiv version^17^ from a supplemental file labeled "SNPs of interest tagging genes for functional inferences and networks analysis." Using Bedtools v2.29.0,^66^ we searched for overlaps between the location of PD risk SNPs and ATAC-seq peaks from iPSC-derived and primary microglia. The intersecting regions were then evaluated in IGV v2.16.2 for SNPs that overlapped or were nearby (within 100 bp) of an H3K37ac ChIP-seq peaks from primary microglia. In this analysis, we found a total of 73 SNPs that we then ranked by GWAS p-value (Table S1 and related Table 1). The locations of the 73 SNPs in ATAC-seq peaks were annotated using ChIPseeker v3.11.^67^

#### CRISPR/Cas9 deletion in iPSCs

The PGP1 cell line was used to create edited cell lines. CRISPR guides were designed using Benchling. Guide sequences are as follows (lowercase letters denote the sequences added for ligation into the pSpCas9(BB)-2A-GFP (PX458) vector from Addgene. See key resources table for guide sequences. For CRISPR/Cas9 editing, we used methods published by the Zheng lab.^68^ To generate plasmids, 1 μl 100 μM forward and 1 μl 100 μM reverse guide sequences were annealed and phosphorylated using 10X T4 Ligase Buffer and 0.5 μl T4 Kinase (PNK) respectively. The reaction was brought up to 10 μl with ddH_2_0 and incubated in the thermal cycler at 37°C for 30 minutes and 95°C for 5 minutes with a ramp down to 25 °C at 5 °C/min and a hold 10°C. The donor plasmid was digested in a 20 μl reaction with 1 μl BbsI, 2 μl PX458 vector, 2 μl Cut Smart Buffer, and 15 μl ddH_2_O. The reaction was then incubated at 37°C for 5-15 minutes. The vector was gel purified using a 0.8% agarose gel with SYBR safe and the QIAquick Gel extraction kit. Guide duplex sequences were ligated with the PX458 vector using 1 μl diluted oligo duplex (1:250), 50 ng of digested vector, 2 μl 10X T4 ligase buffer, 0.2 μl T4 ligase (final amount = 1 weiss U), and ddH_2_O in a final reaction volume of 20 μl. The reaction was incubated at room temperature for 10 minutes. Following incubation, 0.4 μl PlasmidSafe ATP-dependent DNase, 0.8 μl 10 Mm ATP (25mM ATP soln.), and 2 μl 10X PlasmidSafe Buffer was added to the 20 μl ligation reaction and incubated at room temperature for 30 minutes.

The final vector was transformed using Top10 chemically competent E. coli. One to five μl of plasmid was added to single aliquot E. coli, incubated on ice for 30 minutes, heat shocked without shaking at 42°C, and placed on ice for 2 minutes. Pre-warmed S.O.C (250 μl) was added prior to incubating vials horizontally at 37°C for 1 hour at 225 rpm in a shaking incubator. Cultures (25-100 μl) were spread on pre-warmed ampicillin plates (125 μg/mL) and incubated overnight at 37°C. A well isolated colony was inoculated into a culture of 1-5 mL LB containing ampicillin (100 μg/mL) and incubated 37°C with vigorous shaking for 12-16 hours. Plasmids were isolated using QIAprep Spin Miniprep Kit (250).

For nucleofection of plasmids contain CRISPR guides we used Lonza Human Stem Cell Nucleofector Kit 1. Four μg total plasmid (2 μg of each plasmid) was added to 100 μl of nucleofector solution. The nucleofector solution plus plasmid was added to 400,000 pelleted iPSCs. Cells and solution were then added to the supplied cuvette and electroporated on program A23 using an Amaxa Nuclofector II. Cells were then immediately placed in 24-well plates with growth media plus revitacell. Following nucleofection, cells were allowed to grow for 48 hours prior to sorting. GFP-positive cells were sorted using a BD FACSymphony S6 cell sorter into 96-well plates (one cell per well) and expanded for at least 3 passages from the 96-well plate to a 24-well and finally up to a 6-well plate. Each clone was then PCR screened for the deletion (Figure S4A). We chose two separate clones named A11 and H11 (based on their position in the 96-well plate) to move forward with differentiation and RNA-sequencing.

#### RNA-sequencing

RNA was collected using the QIAGEN QIAshredder and RNeasy isolation kit. Collection happened at three time-points across two separate differentiations. Sample names, their collection times, and total number of technical and biological reps can be found in Figure S4B. We did not perform a power calculation prior to this experiment. However, Liu et al. demonstrates that the power (with FDR = 0.05) achieved by sequencing to a depth of 30M reads or above (we used 50M) with 3 or 4 replicates is about 0.75 and 0.85 respectively.^69^ Paired-end reads that had a quality score below 20 and were less than 20 base pairs in length were removed using Trimgalore (see key resources table for citation). Reads were then aligned with STAR^70^ and quality checked using MulitQC.^61^

#### Sequencing of total RNA-seq libraries

Libraries were prepared by the Van Andel Genomics Core from 10 ng of total RNA using Takara SMARTer Stranded Total RNA-Seq Kit v3 Pico Input Mammalian per the manufacturer’s protocol. In brief, RNA was sheared to 300-400 bp, after which dscDNA was generated using a template switching mechanism, and unique dual indexed adapters were added to each sample. Ribosomal cDNA was degraded by scZapR and scrRNA probes, and libraries amplified with 13 cycles of PCR. Quality and quantity of the finished libraries were assessed using a combination of Agilent DNA High Sensitivity chip, QuantiFluor® dsDNA System, and Kapa Illumina Library Quantification qPCR assays. Individually indexed libraries were pooled and 100 bp, paired-end sequencing was performed on an Illumina NovaSeq6000 sequencer, to return a minimum read depth of 50M read pairs per library. Base calling was done by Illumina RTA3 and output of NCS was demultiplexed and converted to FastQ format with Illumina Bcl2fastq v1.9.0

### Quantification and statistical analysis

Statistical analysis for differential gene expression in microglia was done using edgeR version 3.18.^71^ Comparisons were made by grouping all technical and biological replicates of the edited microglia (A11 and H11, n = 4) and all replicates of the wild-type microglia (WT, n = 3), with a batch correction to account for separate differentiations. For a detailed account of biological and technical replicates see Figure S4B. Using edgeR, we first performed TMM normalization of libraries. Significant differences were then determined using the genewise negative binomial generalized linear model (glmQLFit). Expression levels were considered statistically significant if the FDR value was ≤ 0.05. EdgeR estimates dispersion from replicates using the quantile-adjusted conditional maximum likelihood method (qCML). Details of the statistical analysis can be found in the Figure 3 legend. The individual samples compared can be found in Figure 3B.

## Supplemental information

Document S1. Figures S1–S5

Table S1. GWAS summary stats for 73 candidate SNPs, related to Table 1 and Figure S2

Table S2. MotifbreakR results, related to Figure 2B

Table S3. EdgeR glmfit differential expression results, related to Figure 3
