# Supplementary material for: Parkinson’s disease risk enhancers in microglia
Source: iScience. 2024 Jan 17;27(2):108921. doi: 10.1016/j.isci.2024.108921 (PMC10845915; doi:10.1016/j.isci.2024.108921)
Supplement: Document S1. Figures S1–S5 [file mmc1.pdf]

iScience, Volume *27*

Supplemental information

Parkinson's disease risk enhancers in microglia

Alix Booms, Steven E. Pierce, Edwin J.C. van der Schans, and Gerhard A. Coetzee

Supplemental figures, titles, and legends

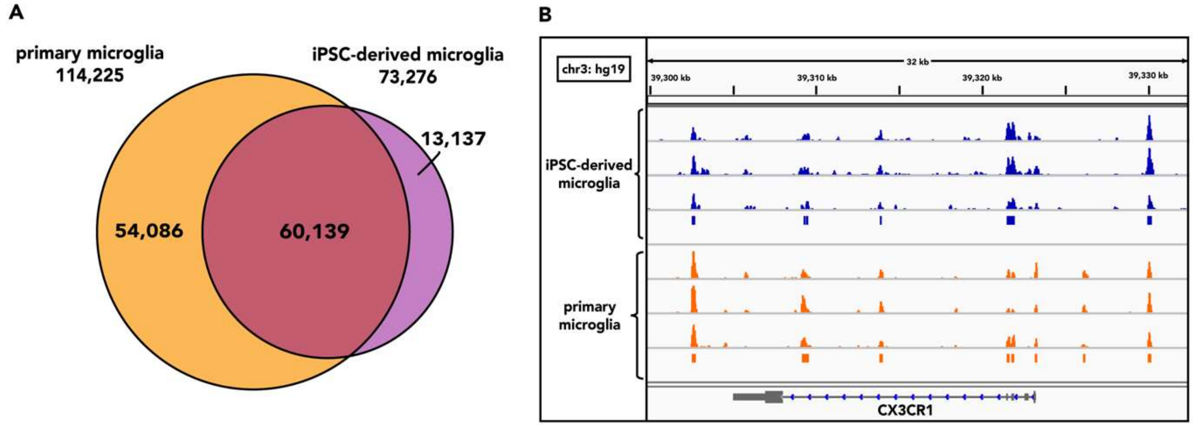

Figure S1. Comparison of ATAC-seq peaks in iPSC-derived microglia to primary microglia, related to STAR Methods

A. Euler diagram of the overlap between primary and iPSC-derived microglia ATAC-seq peaks.
B. IGV screenshot of 3 replicates of the ATCC iPSC-derived microglia cell line (blue) and three different primary microglia cell lines (one replicate of each) (orange) at the CX3CR1 locus. Blue and Orange bars represent peaks called by MACS2 [1].

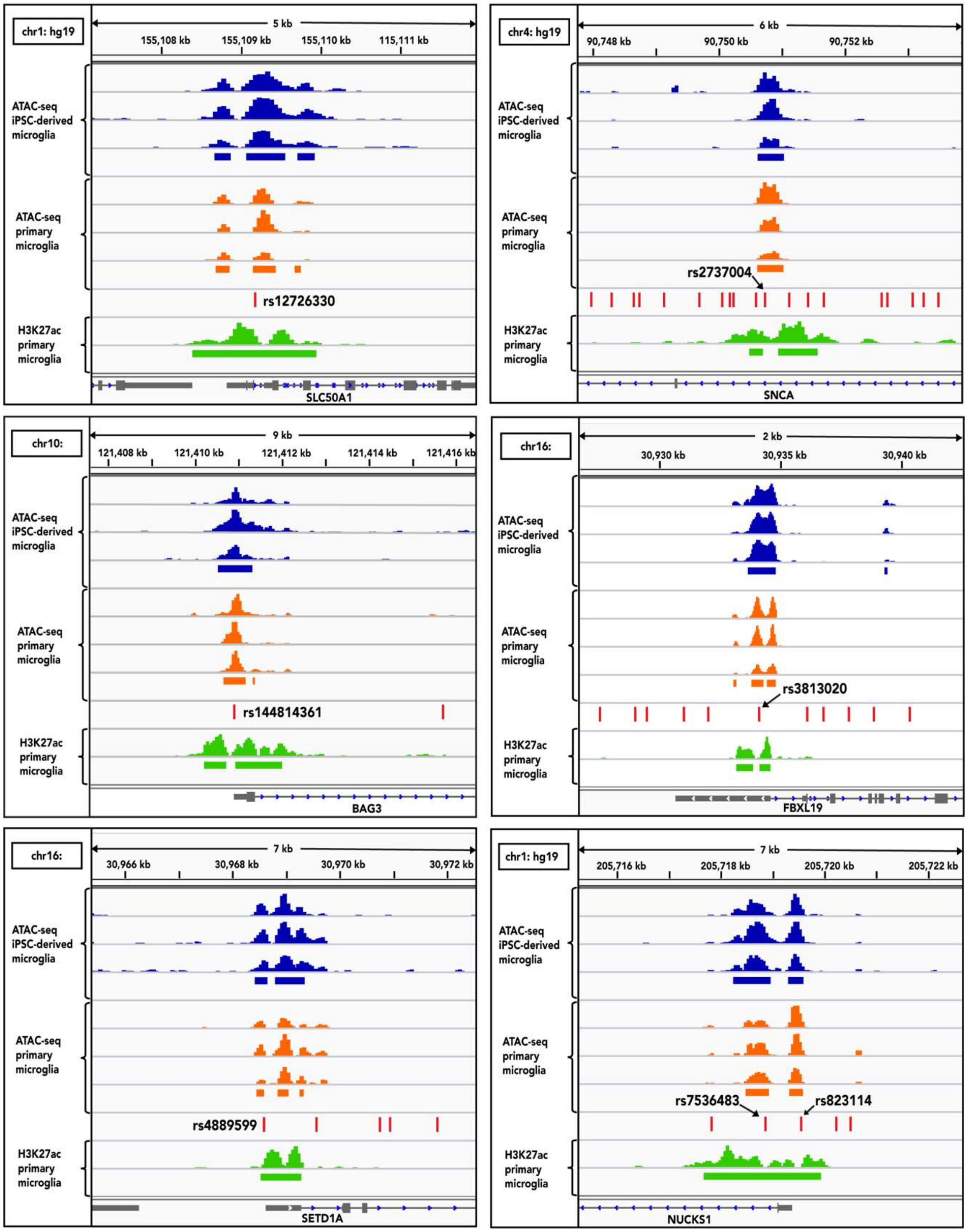

Figure S2. Six top candidate risk SNPs, related to Table 1

Images from Integrative Genomics Viewer (IGV) of top candidate risk SNPs (red lines marked with arrows). These SNPs overlap ATAC-seq peaks from iPSC-derived microglia (blue) and primary microglia (orange). They are also surrounded by H3K27ac histone marks (green).

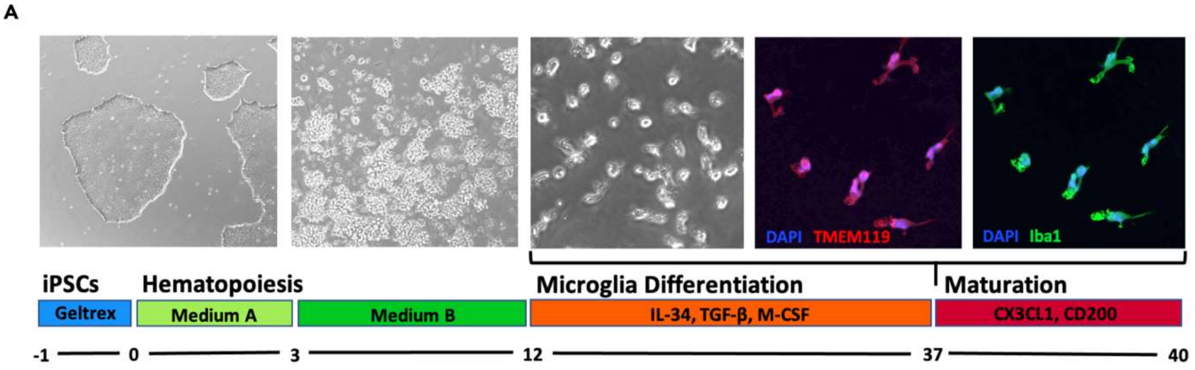

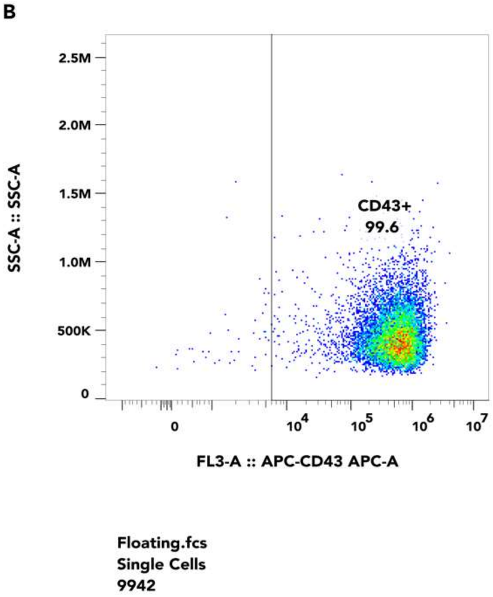

Figure S3. iPSC-derived microglia differentiation process and markers for ATCC cell line, related to STAR Methods

A. Starting from iPSCs on day 0, microglia were differentiated into hematopoietic progenitors (HPCs) over 12 days. After an additional 16 days (40 days total), cells are fully mature and stained for microglia markers Iba1 and TMEM119.
B. FLOW results showing 99.6% of HPCs are CD43+ on day 12 of differentiation.

A

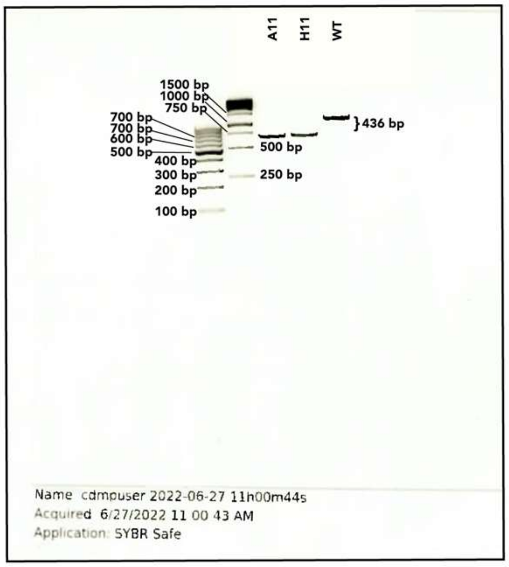

B

**Differentiation 1**			
**stage**	**iPSCs**	**HPCs**	**Microglia**
	**A11ipsc_1**	**A11hpc_1**	**A11mic1_1**
			**A11mic2_1**
	**H11ipsc_1**	**H11hpc_1**	
	**WTipsc1_1**	**WThpc1_1**	**WTmic1_1**
	**WTipsc2_1**	**WThpc2_1**	**WTmic2_1**
**Differentiation 2**			
**stage**	**iPSCs**	**HPCs**	**Microglia**
	**A11ipsc_2**	**A11hpc1_2**	**A11mic2_2**
		**A11hpc2_2**	
	**H11ipsc_2**	**H11hpc1_2**	**H11mic2_2**
		**H11hpc2_2**	
	**WTipsc_2**	**WThpc1_2**	**WTmic_2**
		**WThpc2_2**	
**Total Replicates**			
	**iPSCs**	**HPCs**	**Microglia**
**A11**	**2**	**3**	**3**
**H11**	**2**	**3**	**1**
**WT**	**3**	**4**	**3**

Figure S4. Validation of enhancer deletion and replicate table, related to Figure 2 B and Figure 3

A. Gel image showing the PCR product of wild-type (WT) and enhancer deletion clones (A11 and H11) using primers that flank the deletion site.
B. Table showing the sample names, collection times, and total number of replicates.

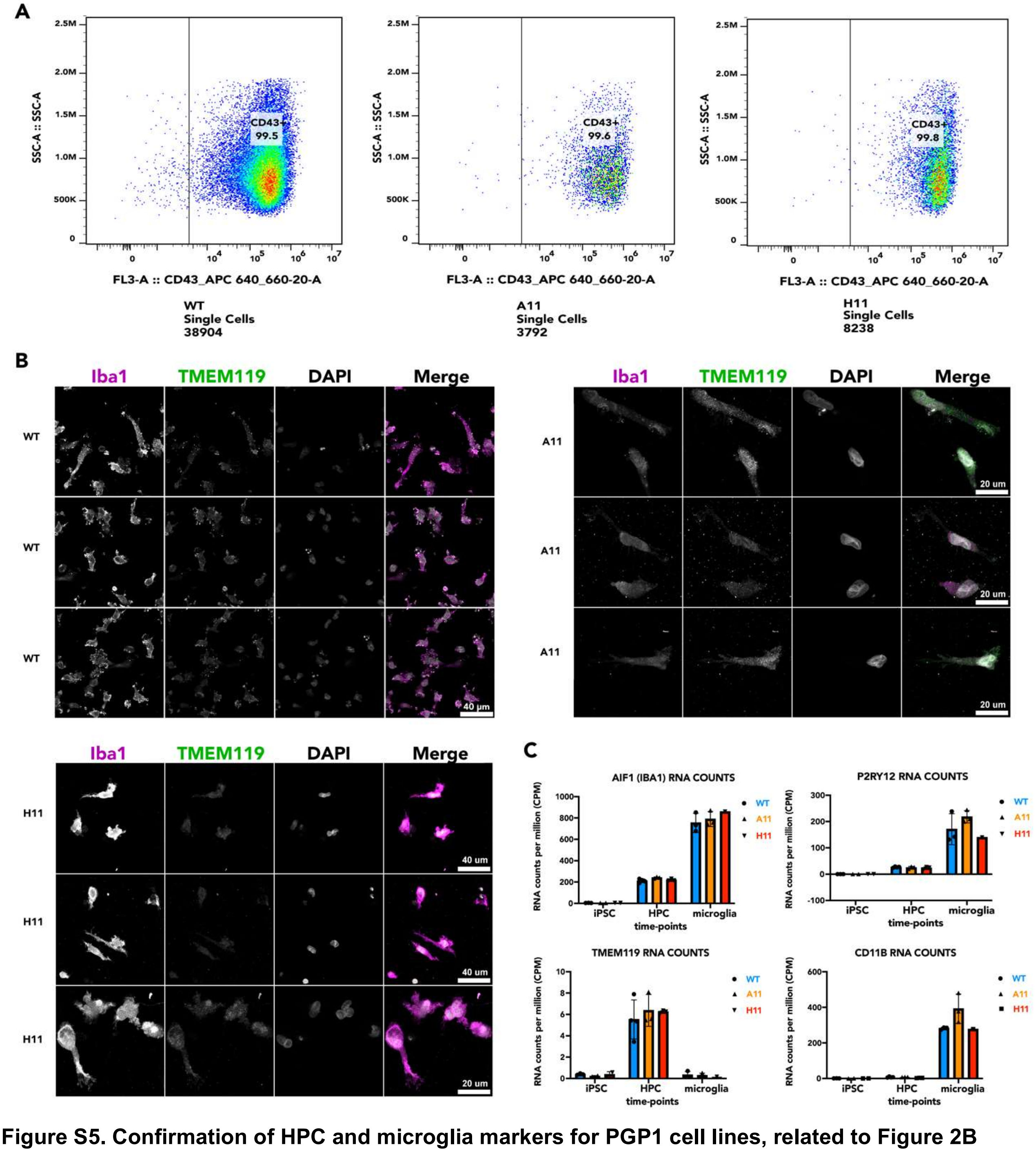

Figure S5. Confirmation of HPC and microglia markers for PGP1 cell lines, related to Figure 2B and Figure 3

A. FLOW results to quantify the percentage of CD43+ HPCs at day 12 of differentiation for WT, A11, and H11. All cell lines were over 95% positive for CD43.

B. Average intensity z-projection of images showing Iba1 and TMEM119 expression in WT, A11, and H11 cell lines. Any images larger than 1012x1012 pixels were cropped for uniformity. Scale bars range are either 20 or 40 μM.

C. RNA counts per million (CPM), adjusted using TMM normalized library sizes, of microglia marker genes. No significant differences were observed between cell lines (WT = 3 and enhancer

deletion = 4 (A11 = 3 + H11 = 1) for *AIF1*, *CD11B*, *P2RY12*, and *TMEM119* as determined by edgeR Benjamini Hochberg corrected FDR values. Error bars represent the standard deviation.

SUPPLEMENTAL REFERENCES

1. Zhang, Y., Liu, T., Meyer, C.A., Eeckhoute, J., Johnson, D.S., Bernstein, B.E., Nusbaum, C., Myers, R.M., Brown, M., Li, W., and Liu, X.S. (2008). Model-based analysis of ChIP-Seq (MACS). Genome Biol 9, R137.
